# Challenges in providing maternity care in remote areas and islands for primary care physicians in Japan: a qualitative study

**DOI:** 10.1186/s12875-018-0806-6

**Published:** 2018-07-18

**Authors:** Ayako Shibata, Makoto Kaneko, Machiko Inoue

**Affiliations:** 1 Department of Obstetrics and Gynecology, Yodogawa Christian Hospital, 1-7-50, Kunijima, Higashiyodogawa-ku, Osaka, 533-0024 Japan; 2 Musashikoganei Clinic, Japanese Health and Welfare Co-operative Federation, 1-15-9, Honcho, Koganei-shi, Tokyo, 184-0004 Japan; 3 Division of Clinical Epidemiology, Jikei University School of Medicine, 3-25-8, Nishishimbashi, Minato-ku, Tokyo, 105-8461 Japan; 4 Department of Family and Community Medicine, Hamamatsu University School of Medicine, 1-20-1, Handayama, Higashi-ku, Hamamatsu, Shizuoka, 431-3192 Japan

**Keywords:** Maternity care, Primary care, Qualitative study, Rural medicine, Women’s health

## Abstract

**Background:**

Maintaining a maternity care system is one of the biggest issues in Japan due to the decreasing number of obstetricians, especially in remote areas and islands. The aim of this qualitative study was to explore the challenges in women’s health and maternity care in remote areas and islands for primary care physicians and obstetricians in order to provide an insight necessary to develop a better health care system.

**Methods:**

We conducted semi-structured interviews with 13 primary care physicians and 4 obstetricians practicing maternity care at clinics/hospitals in remote areas and islands across Japan. Interview data were analyzed, using the modified Grounded Theory Approach, to elucidate the challenges primary care physicians faced in their practice.

**Results:**

Primary care physicians who engaged in maternity care recognized the following challenges: low awareness of primary care, lack of training opportunities, unclear goal of the training, lack of certification system, lack of consultation system, and lack of obstetricians to offer support. These six challenges along with the specialty’s factors such as sudden changes of patients’ condition were considered to result to the provider’s hesitation and anxiety to engage in the practice.

**Conclusions:**

This study found six environmental/systemic factors and three specialty’s factors as the main challenges for primary care physicians in providing maternity care in remote areas and islands for primary care physicians in Japan. Increasing the awareness of primary care and developing a maternity care training program to certify primary care physicians may enable more primary care physicians to engage in and provide women’s health and maternity care in remote areas and islands.

## Background

The Japanese maternal mortality rate is as low as 4 in 100,000 deliveries; most of those deliveries are covered by obstetricians [1]. However, obstetricians are decreasing in number, creating difficulties in supporting the perinatal care needs of remote areas [2]. The number of new members of the Japan Society of Obstetrics and Gynecology (JSOG) decreased for 3 years straight from 2010 with approximately 24% of members being 65 years old or older [3]. Sixty percent of members aged 30 years old or younger are female physicians [3]. The number of obstetricians working at maternity facilities is expected to decrease going forward. Furthermore, the number of new obstetricians per capita in Japanese prefectures shows marked disparity (expected to increase going forward) between Tokyo (4.51 obstetricians per 100,000 residents) and Fukushima and Ibaraki (0.75 obstetricians per 100,000 residents) [3]. The JSOG has moved forward to concentrate on obstetrics facilities aiming to reduce the burden of obstetricians. This had decreased the number of obstetric hospitals by 20% between 2005 and 2011 [4].

Currently, there are several areas where access from delivery institutions to perinatal medical centers takes 60 min or longer, primarily in Kyushu, Tohoku, Sanyo, Shikoku, and the outer islands, which have keenly experienced the decrease or absence of obstetricians [5]. We must consider how and where to carry out regional maternity care and who will carry out that care.

Some other countries assign primary care physicians (PCPs) to handle low-risk maternity care. Those systems coordinate obstetricians and PCPs especially in areas where obstetricians are scarce. For example, in the U.S., perinatal care, including child delivery, is a required part of specialized training for PCPs (family practitioners). The addition of a one-year obstetrical fellowship allows for PCPs to practice Caesarean section operations. Boston University Municipal Hospital is trying to create a joint maternity care team of obstetricians and PCPs [6]. In Canada, although the percentage of PCPs practicing child delivery has fallen to 11.1%, the percentage of PCPs offering maternity and postpartum care remains high at 50% [7]. Some PCP clinics are trying to focus on perinatal and maternity care [8]. In Australia, a collaborative system called “shared maternity care” was built to coordinate regional PCPs’ clinics/maternity centers and high-volume perinatal medical centers handling child delivery [9].

The current training program for Japanese PCPs was accredited by the Japan Primary Care Association (JPCA) [10], which is an academic organization of PCPs established in 2010. It mandated 3 months of pediatric training in addition to 6 months of internal medicine training in 3 years, but training in obstetrics and gynecology (OBGYN) is elective [11]. Currently, places where PCPs offer maternity care are limited to only a handful of remote areas and islands across Japan. Narumoto has surveyed three of these areas where obstetricians and PCPs are collaborating to offer maternity care, showing that the collaboration reduces the burden of the obstetricians and allows them to focus on more specialized care [12]. The JSOG stated the need to consider dividing obstetrician role with primary care physicians in the grand design of renovation of the OBGYN healthcare system (GD2015) [13]. It is possible that Japan will need PCPs to handle some women’s health and maternity care in near future. However, the discussion on this issue has not progressed much, and no specific strategies for role sharing have been decided. This qualitative study aimed to clarify the challenges for PCPs and obstetricians in women’s health and maternity care in remote areas and islands in order to provide an insight necessary to develop a better health care system.

## Methods

**Fig. 1:**
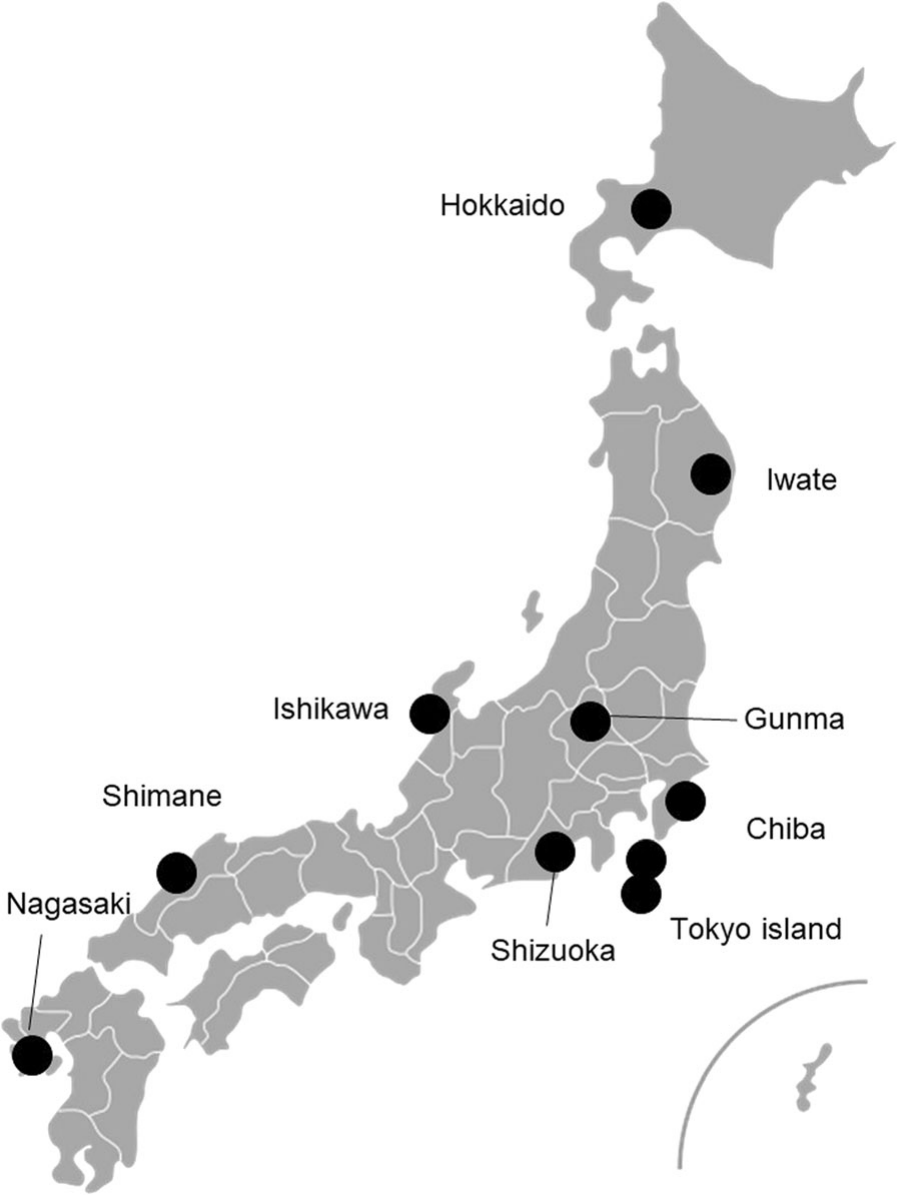
Places in Japan where research participants work. Map image is Creative Commons CC0 1.0 Universal Public Domain Dedication

We conducted qualitative research using semi-structured interviews. We recruited the participants from the physicians who practiced women’s health and maternity care in the remote areas and islands in the member of the JPCA. We included both residency program directors, and residents in order to obtain broader perspectives. We sent e-mails with the research protocol and asked voluntary participation to the study. The first author visited the participants for interview who had agreed to participate. Research participants were 17 doctors (4 obstetricians and 13 PCPs) offering women’s health and maternity care in the remote areas and islands: Hokkaido, Tohoku, Ishikawa prefecture, Tokyo, Chiba prefecture, Shizuoka prefecture, Shimane prefecture, and Nagasaki prefecture (Fig. 1).

The interview guide was created through the discussion among the researchers, one of whom had an experience of solo practice in a remote island. Interview questions focused on the following: (1) the types of women’s health and maternity care offered by PCPs, (2) the challenges they faced in providing that care, and (3) what would be necessary for PCP–obstetrician collaboration (Appendix 1).

Participants were informed ahead of time of the voluntary nature of survey participation and their freedom to withdraw from participation. Interviews were conducted in private in separate rooms or meeting facilities between April of 2015 and March of 2016. After consent forms were received, interviews were audio-recorded. The interviewer used interview guide along with open-ended questions in order to collect wide range of participants’ opinions and feelings. Written consent was obtained from all participants prior to data collection. This research conformed to the provisions of the Declaration of Helsinki and was approved by the ethics committee of Yodogawa Christian Hospital.

The recordings were transcribed verbatim, and the identifying information was removed. The modified version of Grounded Theory Approach (m-GTA) was used for data analysis [14], which had been developed by Kinoshita on the basis of the grounded theory approach, with following procedure [15]. With m-GTA, the data are not segmented by predefined unit of a word or a sentence as in the grounded theory approach, but by a unit of sentences that allows deeper interpretation of meaning. As we reviewed the transcripts, we extracted concrete examples which reflected the participants’ specific views on the challenges they faced, and we shaped concepts derived from these pieces of data by repeatedly interpreting the meaning. Throughout the procedure, we developed an analysis worksheet for each concept with concept name, definition, and concrete examples. We iteratively conducted the analysis and generated new concepts while comparing and examining the examples obtained from new data. We continued the data collection until the point when theoretical saturation was reached where no more new concepts had emerged. Next, we examined the concepts, and by grouping some concepts, deliberated on the categories that represented the challenges PCPs face in providing women’s health and maternity care in remote areas and islands. Then, we created a conceptual model by interpreting the interrelationships among the final concepts and categories. All researchers held discussions to agree upon the development of concepts, categories and the conceptual model. We did not use any software for coding.

## Results

PCPs in this study included general internists, family practitioners, and senior residents of family practice who engaged in primary care in remote areas and islands. Of the 17 participants, 4 were obstetricians, and 13 were PCPs (with 1 holding obstetrical certification and 2 undergoing obstetrical training); 15 were men, and 2 were women. Six participants were graduates of Jichi Medical School (which requires graduates to work 9 years in remote areas). Four participants are presently offering practice on remote islands; 3 had experience with remote island practice. Participant backgrounds are shown in Table 1. The average interview time was 65 min (ranging from 32 to 101 min).

**Table 1:** Backgrounds of research participants

No.	Region	Specialty	Position	Training	Sex
1	Gunma	OB/GYN^a^	Attending	Jichi^b^	M
2	Shimane	PCP^c^	Attending	Jichi, Island practice OB/GYN residency training	M
3	Ishikawa	PCP	Resident	OB/GYN residency training	F
4	Ishikawa	OB/GYN	Attending	PC residency training	M
5	Ishikawa	OB/GYN	Attending	OB/GYN attending	M
6	Ishikawa	PCP	Attending	PCP attending	M
7	Nagasaki	PCP	Resident	OB/GYN residency training	M
8	Tokyo island	PCP	Attending	Jichi, Island practice	M
9	Tokyo island	PCP	Resident	Jichi	M
10	Tokyo island	PCP	Resident	Jichi, Island practice	M
11	Tokyo island	PCP	Resident	Jichi, Island practice	M
12	Shizuoka	PCP	Attending	PCP attending	M
13	Shizuoka	PCP	Resident	OB/GYN residency training	F
14	Iwate	OB/GYN	Resident	OB/GYN residency training	M
15	Hokkaido	PCP	Attending	Island practice	M
16	Hokkaido	PCP	Resident	PCP residency training	M
17	Chiba	PCP	Attending	PCP attending	M

^a^*OB/GYN* Obstetricians/Gynecologist

^b^*Jichi* A graduate of the Jichi University

^c^*PCP* Primary care physician

Three types of women’s health and maternity care were offered by the PCPs in Japan. The first type focused on common gynecological disease (office gynecology). The scope of the practice included vaginal discharge, menstrual abnormalities, conception check, menopausal symptoms, cervical cancer screening. The physicians’ training period was several weeks in an OBGYN department in addition to the outpatient training at a primary care (PC) clinic. This type of practice was observed in the outer Okinawa islands and PC clinics in the Tohoku area. The second type was office gynecology and prenatal care (not including child delivery). The scope of the practice included common gynecological diseases and prenatal care up to the third trimester. The physicians’ training period was 2–3 months outpatient/ward training in an OBGYN department in addition to outpatient training at a PC clinic. This practice was observed in the outer islands and PC clinics in the Kanto area. The third type was comprehensive women’s health care including child delivery. The scope of the practice included office gynecology, child delivery, and practice in an OBGYN ward including assistance with C-sections. The physicians’ training involved 2–3 months of outpatient/ward training and night duties in an OBGYN department and outpatient training at a PC. This style was observed in PC clinics and hospitals in the Chubu region.

**Fig. 2:**
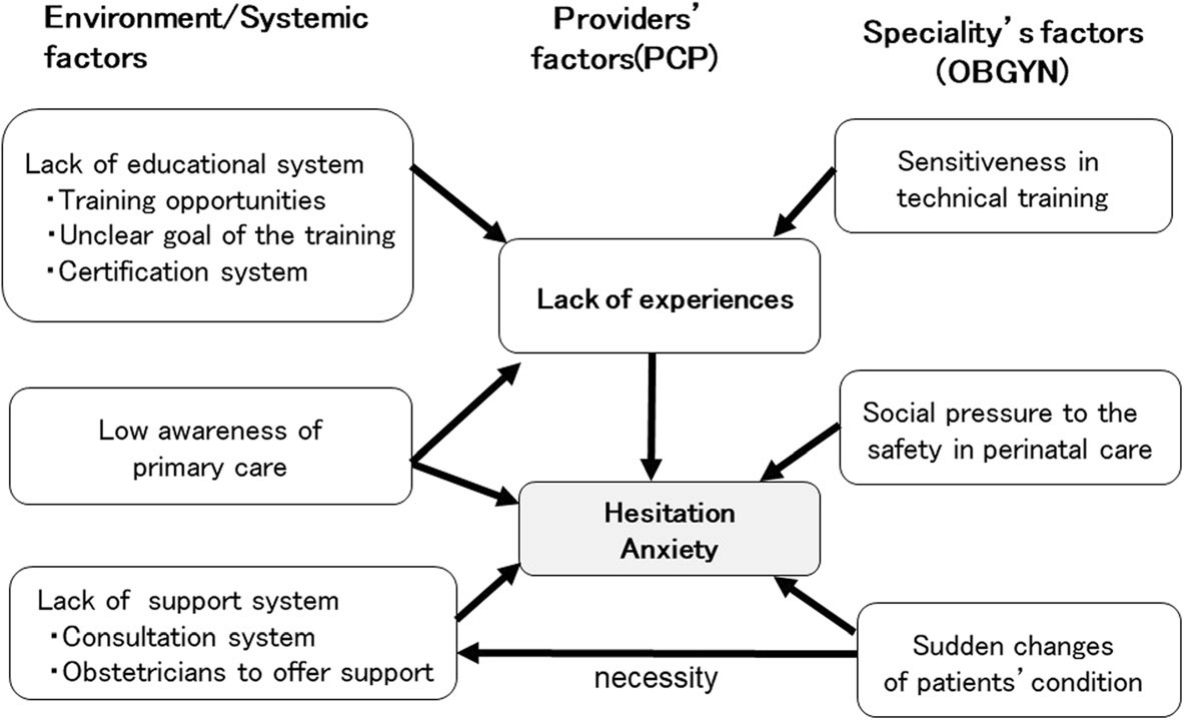
Difficulties for primary care physicians to practice maternity care Conceptual diagram of six environmental/systemic factors and three specialty’s factors faced by primary care physicians handling women’s health and maternity care in Japan

We found following six main challenges faced by PCPs handling women’s health and maternity care in Japan (Fig. 2): (1) Low awareness of primary care; (2) Lack of training opportunities; (3) Unclear goal of the training; (4) Lack of certification system; (5) Lack of a consultation system; and (6) Lack of obstetricians to offer support. The six challenges are assembled into three categories; ‘Low awareness of primary care,’ ‘Lack of educational system,’ and ‘Lack of support system.’ There were also three specialty factors which made difficult for PCPs to engage in maternity care; (1) Sudden changes of patients’ condition; (2) Social pressure for safety in perinatal care; and (3) Sensitivity in technical training. ‘Low awareness of primary care,’ ‘Lack of educational system,’ and “Sensitivity in technical training” were considered to result in lack of experiences of PCPs. ‘Low awareness of primary care,’ ‘Lack of support system’ and lack of experiences were considered to lead to the hesitation and anxiety of the PCPs to engage in handling women’s health and maternity care in remote areas and islands in Japan (Fig. 2). Followings are the explanations of each concept with quotes. Quotes are shown in italics. “PCP” stands for primary care physician, and “OB” stands for obstetrician.

### Low awareness of primary care

The awareness of the concept of “primary care” in patients and medical staff (especially in obstetricians) is low. The Japanese society has a poor understanding of the family physicians and general physicians who take care of women’s health and pregnancy management.

*The majority don’t know how much to entrust to PCPs. (15: PCP)*

*Obstetricians don’t know what we (family practitioners) are nor how to utilize us. They don’t see our faces and don’t know what training we’ve received. So, they worry about us in managing pregnancies and child deliveries where they know anything could potentially happen. (12: PCP)*

*We don’t know the extent to which family practitioners are seeing pregnant patients. (5: OB)*

*Patients seem to wonder whether a family practitioner can provide maternity practice, and I don’t have time to explain this to them. To refer a patient to the family practitioner, I have to explain what a family practitioner is, which is not a simple task. (5: OB)*

### Lack of training opportunities

There are few educational programs in which PCPs can acquire skills and experiences in women’s health and maternity care. Insufficient experience leads to hesitation and anxiety of the PCP in the practice.

*I do have concerns that something could be overlooked in fetal echo. (3: PCP in obstetricians training)*

### Unclear goal of the training

In Japan, educational goals for the training for PCPs to learn women’s health and maternity care are lacking in primary care training. There is no consistency in the scope and content of the practice offered by family practitioners, family doctors, and primary care doctors in Japan.

*Rotating the obstetrics and gynecology department of a big hospital, it tends to be just holding the retractors in the surgery. (16: PCP)*

*If I extend the scope of my (family medicine) clinic practice too much, I can’t imagine what’s going to be. (3: PCP)*

### Lack of certification system

Except for obstetrical board certification, there is no certification system for PCPs to demonstrate their skills and knowledge of women’s health and maternity care in Japan. Some PCPs chose to acquire obstetrical board certification, which requires additional 3 years of training.

*When thinking about the social pressure for the medical safety, including risks for lawsuits, you must protect yourself in a form to take the obstetrical certification (12: PCP)*

### Lack of consultation system

When a PCP practices women’s health and pregnancy management, a supportive facility is needed that accepts consultations and transportation of serious patients. However, there is no consultation system in which PCPs in remote areas and islands can consult with obstetricians when necessary.

*There are some obstetric or gynecological emergencies in a certain probability. We have survived by consulting them to the local obstetric institution. (8: PCP)*

*I’m wondering what would happen if maternity care were only carried out by a family practitioner. It would be good if we have the mother visit a large hospital for a fetal screening as a risk management, or we can discuss with the midwives and the obstetricians on several occasions. (13: PCP in obstetricians training)*

### Lack of obstetricians to offer support

The obstetricians themselves are considered very busy and cannot afford to support PCPs in remote areas.

*There are few obstetricians in the remote areas. (4: PCP)*

*Obstetricians in the university are full of research and their tasks, and it is hard to think of supporting PCPs. (7: OB)*

Additionally, we found three specialty’s factors affecting why obstetrical practice is difficult and challenging for other medical specialties:

### Sudden changes of patients’ condition

The patients’ condition can change rapidly during child delivery, which sometimes requires rapid consultation and patient transfer.

*Some of my patients have become threatened with premature labor or extreme leg edema from preeclampsia. Since there is the possibility of sudden, high-risk fluctuations, we really need the consultation of the obstetricians when taking care of the deliveries. (3: PCP in obstetrical training)*

### Social pressure for safety in perinatal care

There are liability and lawsuit risks surrounding the perinatal care and child delivery.

*I worry about fetal malformations of my patients since we can’t provide fetal screening echo. I have few experiences of fetal malformations such as cardiac deformities. (10: PCP)*

### Sensitivity in technical training

It is considered difficult to teach trainees in the perinatal visit or child delivery, since the patients request not to be seen by trainees.

*It is hard to teach while a patient’s legs are open. (14: OB)*

*Teaching by whispering will cause the patient anxiety. (15: OB)*

The factors described above (six challenges with three specialty’s factors) were considered to result in the provider’s hesitation and anxiety to engage in the women’s health and maternity care. On the other hand, there were many positive comments on having PCPs in maternity care as follows.

*My patients have said, “If my family doctor could deal with gynecological problems, then I’d talk those issues to the doctor.” Some patients are reluctant to visit obstetricians because of the hesitation of vaginal exam. We should probably start from this very modest approach. (7: PCP with obstetrical certification)*

*If we allow PCPs to attend the child delivery, they can also take care of their immunizations later. The mother can also benefit from PCPs, such as annual medical checkup and flu shots. If the mom has any complaints during pregnancy, such as skin issues, asthma,* etc.*, PCPs can take care of it right away without referring her to another doctor. (13: PCP in obstetrical training)*

*Having a PCP join in our night shifts makes a huge difference in terms of the limited number of obstetricians. (5: OB)*

*The merits for obstetricians would be easy to ask PCPs to take care of common complains, psychological care, and postpartum care. (3: PCP)*

*The role of PCPs is to enable specialists to concentrate on the areas their skills are needed in. Hence, the system offers mutual benefits. The patient could have all-around care from the PCPs, and there are cost benefits as well. (7: PCP with obstetrical certification)*

## Discussion

This research examined the practice styles of PCPs who are offering woman and maternity care and challenges they faced in providing that care. The practice styles include (1) office gynecology (care focusing on common women’s health), (2) office gynecology and prenatal care (not including child delivery), and (3) comprehensive women’s health care including child delivery. In Japan, practice style (1) and (2) are commonly seen, and few PCP handles child delivery on their own. These practice styles appear to depend on the number of child births in that region and the presence or absence of obstetricians to support PCPs. In regions where PCPs are managing the child delivery, there are collaborative teams of PCPs and obstetricians in the same facility. If we consider PCPs to manage partial obstetrical practice in the remote area, it is necessary to discuss which practice style is desired and to build training programs and goals accordingly.

Three categories of the six environmental/systemic factors for PCPs providing women’s health and maternity care were low awareness of primary care, lack of educational system and lack of support system. These three factors impede PCPs from gaining sufficient practice experiences, which leads to their hesitation and anxiety in providing the care. First, the low awareness of primary care is a unique problem for Japan. In Japan, there are many names for “general practitioner”: family practitioner, primary care doctor, general practitioner, general internal medicine, hospital generalist, home doctor, and so on. There is no consistency in the definition and content of their practice. There are few people, including patients, obstetricians, and health care professionals, who fully understand the practice offered by each “general practitioner”. Hence, obstetricians feel uneasy in referring their patients and need extra time to explain to the patients, which are all obstacles to collaboration. To alleviate these concerns, we must clarify which PCP has the competency to practice women and maternity care through a certification system that verifies the skills and knowledge attained.

The second factor, “lack of educational system” causes the insufficient experiences of PCPs as women’s health and maternity care providers. Various foreign countries have handled these challenges by developing systems for PCPs to collaborate with obstetricians. For instance, Boston University has tried including the family medicine residents in their maternity care teams, including rounds, night shifts, and perinatal/post-natal care. Bringing family practitioner residents onto these teams gives them exposure to over 40 deliveries a year and increases their practice experience [6]. These collaborative teams could help PCPs to increase maternity care experiences in a protected learning environment.

The third factor, “lack of support system” such as consulting system to the obstetricians. In our interview, some PCPs mentioned the fear of possibly overlooking abnormalities in fetal echo during perinatal checks. At present, the prenatal care guidelines by the JSOG do not mandate fetal screening echo to detect abnormalities [16], although PCPs desire to secure the quality of care by creating the PCP-obstetrician collaboration system. For instant, Australia has a shared maternity care system in which high-volume perinatal medical centers cooperate with regional facilities (PC clinics and maternity centers). Certified facilities can offer perinatal care (at 7–12, 12–14, 22–26, 33, 38, and 39 weeks) by midwives and family doctors [9]. There are clear guidelines for the practice to be carried out at each gestational week as well as criteria for referrals and transfers, which serve to standardized care. These guidelines could help guarantee the quality of care provided, including care offered through PCP–obstetrician collaboration. Japan also has semi-open/open systems in which obstetrical care centers and local obstetrical clinics and midwifery homes collaborate. There are 43 hub obstetrical facilities and 675 collaborating clinics in Japan [17]. However, there are no open manuals for the semi-open/open system, which makes it hard for PCPs to join the system. In the future, collaboration between PCPs and obstetricians might be needed with expanding these semi-open/open systems to the regional PC clinics.

In addition, there were three specialty factors related to obstetrical practice; “Sudden changes of patients’ condition,” “Social pressure to the safety in perinatal care,” and “Sensitivity in technical training.” There are risks of sudden change in condition such as the need for a C-section due to fetal distress or disseminated intravascular coagulation due to postpartum hemorrhage. Therefore, obstetrical care, in contrast to other care, requires a system that allows PCPs to immediately consult with an obstetrician or transfer sick patients to the acute care facilities.

As for social pressure in Japan, there have been multiple cases of medical lawsuits for the accidents in perinatal care, and social pressure is extremely high to ensure the safety of child birth [18]. The Japan Council for Quality Health Care created The Japan Obstetric Compensation System for Cerebral Palsy in 2009 [19]. However, this system is only for the member facilities, which means PCP clinics which do not participate in this system cannot obtain this compensation when medical accidents occur. Recently, the patients have become extremely intent on having the specialist care. The trend is quite strong to request for a specialized physician from the earliest stages of pregnancy and refuse trainee attending. It is getting difficult to provide trainees, including PCPs, with vaginal exam and perineal suture during the training program. From fiscal year 2010, revised residency training systems have started in Japan. Since obstetrical training is not mandatory in 2-year intern training, current residents do not have the opportunity to study child delivery and vaginal exam. To overcome the difficulty of teaching vaginal exam and perineal suture in the clinical settings, we could develop simulations and hands-on training before onsite instruction.

Regardless of these challenges, we have discovered the benefits of involving PCPs in the women and maternity care. Involving PCPs allows the patients to cover various issues all in one place, including perinatal care, infant care, vaccines, cancer screening, blood pressure, and diabetes care after discharge from the delivery ward. Some research reported involving PCPs in maternity care team increases patient satisfaction [6, 20], establishing PCP–obstetrician collaboration could contribute to greater patient satisfaction in Japan, too. Narumoto addressed the benefits of PCP–obstetrician collaboration, explaining that this would ease the burdens on the obstetricians (including inpatients care, outpatient care, and ER walk-ins) and contribute to comprehensive care for the patients [12]. Creating more visibility of those benefits of PCP–obstetrician collaboration should contribute to increase the awareness of primary care in the obstetricians and healthcare professionals.

In the U.S., Brown et al. noted that obstacles to PCP–obstetrician collaboration include trust over sharing care, social pressure over the safety of perinatal care, differences in the culture between specialists and generalists, and patient competition [21]. We revealed six challenges and three specialty factors of difficulties for PCPs to handle the women’s health and maternity care in Japan. This is the first research which gathered the on-site opinions who practice women’s health and maternity care in the remote areas and islands in Japan. The results would provide useful insights for building better health care delivery system of women’s health and maternity care in remote areas and islands.

This study has some limitations. Our research mainly sought the viewpoints of PCPs in Japan to reveal the problems of current health care delivery system. It is necessary to conduct further research on the obstetricians’ perspectives to understand a comprehensive picture of the issues and to build a better collaboration system.

In Japan, 95% of childbearing aged women lived within a 30-min distance from the obstetrics facilities in 2011. This ratio would decrease to around 82 to 90% if we concentrated the obstetrics facilities [22]. The JSOG has stated the need of concentrating the obstetrics facilities and PCP–obstetrician collaboration in order to ease the burden of decreasing obsetricians [13]. We must discuss how to reduce the challenges we found in this study for PCPs in practicing women’s and maternity care. Since the shape of PCP–obstetrician collaboration varies by the countries, consideration is needed in creating a new system that fits the characteristics of Japan’s health care.

## Conclusions

This study found six environmental/systemic factors and three specialty’s factors as the main challenges for PCPs in providing maternity care in remote areas and islands for primary care physicians in Japan. Low awareness of primary care, lack of educational system, and lack of support system as well as specialty’s factors were considered to result in PCP’s hesitation and anxiety to engage in the women’s health and maternity care. In Japan, increasing the awareness of primary care in the patients as well as the obstetricians and developing a maternity care training program to certify PCPs may enable more physicians to engage in and provide women’s health and maternity care in remote areas and islands.

## Appendix 1

The semi-structured interview guide

1. Interview introduction

Explanation of the research aim, how the interview data will be used, and voluntary participation rule. Explanation of anonymity and privacy.

2. Background information

Participant’s career, training history and current position were asked.

3. Current practice

Could you tell me about your practice in the point of women’s health care and maternity care?

- How often do you practice women’s health care and maternity care?
- What kind of medical needs do you experience in women’s health and maternity care?

4. The challenges they faced in providing the care

Could you tell me about the challenges in providing women’s health and maternity care?

- How often and in what situation so you feel those challenges?
- Why do you think those challenges happen?
- Where do you think those challenges come from?

5. About the primary care physicians (PCP) – obstetrician collaboration

Could you talk me about your impression about PCP–obstetrician collaboration in Japan?

Could you talk me about your opinion about PCP–obstetrician collaboration from the perspective of providing women’s health and maternity care in remote areas and islands?

What do you think is needed to improve PCP–obstetrician collaboration to provide women’s health and maternity care?

6. For a better system

What do you think of women’s health and maternity care in the remote areas and islands in Japan?

How do you think women’s health and maternity care in the remote areas and islands in Japan can be improved?

What kind of system do you think is needed to improve women’s health and maternity care in the remote areas and islands in Japan?

## Abbreviations

GD2015: The grand design of renovation of the OBGYN healthcare system
Jichi: A graduate of the Jichi University
JPCA: The Japan Primary Care Association
JSOG: The Japan Society of Obstetrics and Gynecology
OB: Obstetrician
OB/GYN: Obstetricians/Gynecologist
OBGYN: Obstetrics and gynecology
PC: Primary care
PCP: Primary care physicians
